# Evaluation of immune and stress status in harbour porpoises (*Phocoena phocoena*): can hormones and mRNA expression levels serve as indicators to assess stress?

**DOI:** 10.1186/1746-6148-9-145

**Published:** 2013-07-17

**Authors:** Sabine Müller, Kristina Lehnert, Henrike Seibel, Jörg Driver, Katrin Ronnenberg, Jonas Teilmann, Cornelius van Elk, Jakob Kristensen, Eligius Everaarts, Ursula Siebert

**Affiliations:** 1 Institute for Terrestrial and Aquatic Wildlife Research, University of Veterinary Medicine Hannover, Foundation, Büsum 25761, Germany; 2 Institute for Coastal Research, Helmholtz-Zentrum Geesthacht, Geesthacht 21502, Germany; 3 Veterinary Clinic, Reinsbüttel 25764, Germany; 4 Departement of Bioscience, Aarhus University, Frederiksborgvej 399, 4000, Roskilde, Denmark; 5 Dolfinarium Harderwijk, Strandboulevard Oost 1, Harderwijk, AB 3841, The Netherlands; 6 Fjord and Belt, Margrethes Plads 1, Kerteminde 5300, Denmark; 7 SOS Dolfijn, Strandboulevard Oost 1, Harderwijk, AB 3841, The Netherlands

**Keywords:** Harbour porpoise, Stress hormones, Cytokines, Anthropogenic impact, Offshore wind farms, Underwater noise

## Abstract

**Background:**

The harbour porpoise is exposed to increasing pressure caused by anthropogenic activities in its marine environment. Numerous offshore wind farms are planned or under construction in the North and Baltic Seas, which will increase underwater noise during both construction and operation. A better understanding of how anthropogenic impacts affect the behaviour, health, endocrinology, immunology and physiology of the animals is thus needed. The present study compares levels of stress hormones and mRNA expression of cytokines and acute-phase proteins in blood samples of harbour porpoises exposed to different levels of stress during handling, in rehabilitation or permanent human care.

Free-ranging harbour porpoises, incidentally caught in pound nets in Denmark, were compared to harbour porpoises in rehabilitation at SOS Dolfijn in Harderwijk, the Netherlands, and individuals permanently kept in human care in the Dolfinarium Harderwijk and Fjord & Belt Kerteminde, Denmark. Blood samples were investigated for catecholamines, adrenaline, noradrenaline and dopamine, as well as for adrenocorticotropic hormone (ACTH), cortisol, metanephrine and normetanephrine. mRNA expression levels of relevant cell mediators (cytokines IL-10 and TNFα, acute-phase proteins haptoglobin and C-reactive protein and the heat shock protein HSP70) were measured using real-time PCR.

**Results:**

Biomarker expression levels varied between free-ranging animals and porpoises in human care. Hormone and cytokine ranges showed correlations to each other and to the health status of investigated harbour porpoises. Hormone concentrations were higher in free-ranging harbour porpoises than in animals in human care. Adrenaline can be used as a parameter for the initial reaction to acute stress situations; noradrenaline, dopamine, ACTH and cortisol are more likely indicators for the following minutes of acute stress. There is evidence for different correlations between production of normetanephrine, metanephrine, cortisol and the expression of IL-10, HSP70 and haptoglobin.

**Conclusions:**

The expression patterns of the selected molecular biomarkers of the immune system are promising to reflect the health and immune status of the harbour porpoise under different levels of stress.

## Background

The harbour porpoise (*Phocoena phocoena*) is the only reproducing cetacean species in the Baltic Sea and eastern North Sea [1-3]. These waters are highly exposed to anthropogenic impacts as an increasing number of human activities are conducted in these areas, including shipping, construction of offshore wind farms, fisheries, seismic and military operations. These activities cause underwater noise which results in disturbance of and stress in the marine fauna [3-9].

High-intensity underwater noise can cause permanent or temporary threshold shifts in the auditory system and, apart from being responsible for masking and behavioural changes, it is supposed to cause an increased level of stress for the animals [4,10]. Stress is often defined as the body’s reaction to a change that requires a physical, mental or emotional adjustment or response. Although stress occurs naturally in wildlife due to e.g. social competition, breeding behaviour or environmental demands, anthropogenic impacts such as vessel traffic, noise, fishing and chemical pollution can impose significant additional stress [10-12]. Capture and handling are critical stress factors especially for wild marine mammals but also for those temporarily or permanently in human care [13-16]. Periods of stress cause endocrinological, immunological and physiological reactions that can result in immune suppression and deleterious health effects if stressors are chronic [11,17,18]. Catecholamines are associated with a fast “fight-or-flight” reaction as their levels increase rapidly in response to stressors [11,19]. Cortisol is an endogenous glucocorticoid produced in the adrenal cortex under control of adrenocorticotropic hormone (ACTH), produced in the pituitary gland which, in turn, is controlled by the hypothalamus (corticotropin-releasing hormone, CRH). It has a broad spectrum of metabolic and immune-mediating effects mainly associated with stress reactions [11,20,21].

Stress-induced changes to the immune system can increase the risk of infections, neoplasia and immune-mediated disorders [11,22,23]. We chose biomarkers for the detection of pathological immune responses in order to determine the impact of anthropogenic stressors on the health status of free-ranging porpoises and individuals in human care. Acute-phase proteins serve diverse physiological functions of the immune system and are used as markers to detect infection and stress [24-26]. The acute-phase protein HSP70 has been reported to be a cell mediator reacting to bacterial, viral and parasitic pathogens [27,28]. Cytokines and acute-phase proteins as immune mediators have been used to assess the health status and immune system in cetaceans and pinnipeds [29-37].

The aim of this study was to measure stress hormones and cell mediators of the stress-related immune response in the blood of harbour porpoises to determine anthropogenically caused stress. Catecholamines (adrenaline, noradrenaline, dopamine) and their degradation products metanephrine and normetanephrine, cortisol, adrenocorticotropic hormone (ACTH), and messenger-Ribonucleic acid (mRNA) expression levels of selected cell mediators responsible for the immune response heat shock protein (HSP70), acute-phase proteins (APP) haptoglobin (HP), C-reactive protein (CRP) and cytokines Interleukin 10 (IL-10) and Tumor Necrosis Factor (TNFα) were studied.

## Methods

### Harbour porpoise sampling

For the present study 28 live porpoises were sampled. Those animals can be divided into three different groups:

(1)  Free-ranging animals (number (n) =7), by-caught in pound-nets by Danish fishermen, were sampled during field trials including tagging and recording of audiograms of the animals.

(2)  Porpoises sampled at the Dolfinarium Harderwijk, the Netherlands, (n = 8) and at the Fjord and Baelt Kerteminde, Denmark, (n = 1) are kept permanently in human care (1–13 years). These animals are trained for medical purposes to allow voluntary handling and sampling but were taken out of the pool for blood sampling in this study.

(3)  Porpoises in rehabilitation (n = 12) at the rehabilitation centre SOS Dolfijn in Harderwijk were found stranded and are in human care only temporarily (3–6 months). During their stay in the rehabilitation centre these animals are getting accustomed to human handling. They were sampled over several weeks at varying frequency depending on the duration of their rehabilitation and on their health status.

### Blood sampling

Blood samples were obtained from the veins of the fluke using the method described by [12]. Handling and sampling of the free-ranging porpoises were carried out directly on board the fishermen’s boats after the harbour porpoises had been lifted out of the pound nets [12], experimental research on the study animals followed internationally recognized guidelines and was approved by an appropriate ethics commttee with permits from the Danish Forest and Nature Agency SN 343/SN-0008 and Ministry of Justice 1995-101-62]. Three samples were taken: the first directly when the animal was taken out of the pound net, the second two and the last 3.5 hours later.

Sampling of the animals in permanent human care and in rehabilitation was performed during routine medical investigations in the facilities. Sampling was conducted directly after the animals were taken out of the water. The broad spectrum of parameters to be analysed required fairly large sample volumes of about 10 ml, but it was not always possible to obtain a sufficient amount of blood. Due to this limitation not every animal was investigated for the full hormone and biomarker profiles.

### Differential haematology and serum chemistry

For differential haematology and serum chemistry, venous whole blood was collected in tubes with ethylenediaminetetraacetic acid (EDTA) anticoagulant and tubes with coagulation gel for serum extraction. Differential haemogram profiles were generated with a ScilVet ABC™ Animal Blood Counter (Scil Animal Care Company GmbH, 68519 Viernheim, Germany). Serum separator tubes were centrifuged for 15 minutes after blood was clotted (Hettich™ EBA I centrifuge, Andreas Hettich GmbH & Co. KG, 78532 Tuttlingen, Germany). Serum was extracted, frozen at −20°C and later sent to the veterinary laboratory Synlab Vet in Geesthacht, Germany, for determination of blood chemistry parameters.

### Hormone analysis

Blood was collected in tubes with EDTA, ethyleneglycoltetraacetic acid (EGTA) anticoagulant and tubes with coagulation gel for serum extraction, cooled until centrifugation at 2500 U/min for 15 minutes, and frozen at −20°C in 1 ml aliquots. Serum, EGTA plasma and EDTA plasma hormone levels were measured following standard techniques in a commercial laboratory (Endokrinologikum, Hamburg, Germany). For the quantitative analysis of the catecholamines adrenaline (41 samples, Firma Recipe Chemicals + Instruments GmbH München, Intra-Assay: CV 7.6%, Inter-Assay: 4.2%), noradrenaline (41 samples, Firma Recipe Chemicals + Instruments GmbH München, Intra-Assay: CV 6.7%, Inter-Assay: 5.3%) and dopamine (41 samples, Firma Recipe Chemicals + Instruments GmbH München, Intra-Assay: CV 6.1%, Inter-Assay: 3.9%) a high-performance liquid chromatography (HPLC) was performed. Cortisol levels (46 samples) were estimated via a competitive electrochemiluminescent immunoassay (ECLIA, Cobas sheep-antibody, Intra-Assay: CV 1.5-1.6%, Inter-Assay: 1.1-1.3%), levels of ACTH (32 samples, CLIA chemiluminescent immunoassay, DiaSorin, monoclonal mouse-antibody, Intra-Assay: CV 2.6-4.9%, Inter-Assay: 5.5-8.9%) by a non-competitive electrochemiluminescent immunoassay. Metanephrine (25 samples, Labor Diagnostic Nord GmbH & Co. KG Nordhorn, LDN, rabbit antiserum, Intra-Assay: CV 8.7-15%, Inter-Assay: 11-14%) and normetanephrine (25 samples, Labor Diagnostic Nord GmbH & Co. KG Nordhorn, LDN, rabbit antiserum, Intra-Assay: CV 7.9-10%, Inter-Assay: 5.6-8.3%) contents were estimated using a radioimmunoassay.

### Real-time PCR analysis

Blood samples were taken in EDTA monovettes containing 3 ml of RNA*later* and ice cooled until arrival at the lab where RNA was isolated. Total RNA was isolated from 500 μL EDTA blood (RiboPure™Blood Kit, Ambion Europe, Huntington, UK) according to the manufacturer’s protocol.

Published primers were used for the housekeeping gene Glyceraldehyde 3-phosphate dehydrogenase (GAPDH) [29], the cytokines IL-10 and TNFα [32] and the APPs CRP and Haptoglobin (HP) [33] and HSP70 [34]. Primers for the housekeeping gene 14-3-3 protein zeta/delta (YWHAZ) were designed and established by [38]. 80–100 ng/μL RNA was reverse transcribed with murine reverse transcriptase (Real time-Polymerase chain reaction (RT-PCR) Core Kit™; Applied Biosystems, Weiterstadt, Germany). The resulting cDNA served as a template for real-time PCR using the Thermocycler MX4000™ Real-Time PCR Systems (Stratagene Europe, Amsterdam, Netherlands). For real-time quantification the Brilliant III Ultra-Fast SYBR® Green QPCR Master Mix (Agilent Technologies) was used, containing SYBR Green I as a fluorescence dye, dNTP’s, MgCl_2_ and a hot start Taq DNA polymerase. For each parameter a standard curve was prepared using a dilution series from 10e8 to 10e2 copies. The polymerase chain reaction (PCR) started with an initial step at 95°C for 3 min, followed by 45 cycles with denaturation at 95°C for 5 sec and a primer-specific annealing temperature for 20 sec. The annealing temperatures are shown in Table 1. Fluorescence was measured at the end of the annealing and at the end of the dissociation program at a wavelength of 530 nm. To exclude measurement of nonspecific PCR products and primer dimers, and to determine true amplification, each PCR was followed by a dissociation program for 1 min at 95°C, followed by 41 cycles during which the temperature was increased in each cycle, starting at 55°C and ending at 95°C. Only PCR reactions with one well-defined peak were used for analysis. All reactions were performed in duplicate. The constitutively expressed housekeeping genes GAPDH and YWHAZ were used as control genes and to normalise cytokine and APP expression. geNorm software (version 3.5; March 2007) was used to test stability of the candidate reference genes [39].

**Table 1:** Primer-specific sequences, annealing temperatures and base pair length for housekeeping genes, cytokines, acute-phase proteins and heat shock protein

**Cellmediators/mol. biomarkers**		**Primer sequences (5′–3′)**	**Annealing temperature**	**Base pair length**
YWHAZ	S	AGA CGG AAG GTG CTG AGA AA	58°C	208
	AS	TCA TVA CCA GCA GCA ACT TC		
GAPDH	S	GCC AAA AGG GTC ATC ATC TC	57°C	201
	AS	GGG GCC ATC CAC AGT CTT CT		
IL-10	S	CCT GGG TTG CCA AGC CCT GTC	62°C	208
	AS	ATG CGC TCT TCA CCT GCT CC		
TNFα	S	GGC TGA ACA CAT ATG CCA AC	57°C	111
	AS	TGA AGA GGA CCT GGG AGT AGA		
HP	S	CTG GCA GGC TAA GAT GGT TT	54°C	75
	AS	GTC AGC AGC CAT TGT TCA TT		
CRP	S	TTC TCG TAT GCC ACC AAG AG	54°C	192
	AS	TTC AGA CCC ACC CAC TGT AA		
HSP 70	S	GGG GCT GAA CGT GCT GAG G	62°C	280
	AS	CCG CTT GTT CTG GCT GAT GTC		

### Statistical analysis

Statistical evaluations were performed with the free statistics software R[R version 2.15.2, [40]]. Linear mixed models (LMM) were applied throughout on log-transformed data using the package “nlme” [41]. For comparisons between the three groups, animals in human care, in rehabilitation and free-ranging, sampling (repeated measures) was included as random slope term, where possible, and the “Porpoise ID” (the identification number of individual porpoises) as random intercept to avoid pseudo-replication. For some hormones and biomarkers (normetanephrine, ACTH, Cortisol, CRP, HP, TNFα), we included sampling as nested factor in the identification (ID) term because of numerical problems due to fewer measurements per animal. We included the group (free-ranging, rehabilitation and human care) and a sex factor as fixed effects. We achieved minimal adequate models of fixed effects by backward selection based on the lowest AIC (Akaikes Information Criterion) values using maximum likelihood estimation. To allow for variance heterogeneity, a variance-by-group structure was included if diagnostic plots and AIC values indicated violation of the residual homogeneity assumption (CRP and TNFα). To test the effect of time for animals in rehabilitation, we chose the first and last received blood sample of those porpoises and compared them to free-ranging animals and animals in human care. The porpoise ID was included as random intercept term. To test relations between hormones and biomarkers we conducted Spearman rank correlations between all hormones and biomarkers. Only relations with a correlation coefficient rho > 0.4 are presented.

## Results

### Differential haematology and serum chemistry

Differential haematology was conducted for seven free-ranging animals, one of them was sampled once, four were sampled twice and two were sampled three times. The number of leukocytes varied between 3,600 and 9,000 cells per cubic millimetre of blood. In comparison to previous levels one animal showed an elevated level of leukocytes indicating an infection. With the exception of one animal, leukocyte levels decreased between 3-11% in animals if measured three times in the same animal around the time an audiogram was measured. No animal showed indications of a stress leukogram compared to reference values (elevated neutrophils in combination with eosinopenia, lymphopenia and occasionally monocytosis; [42]). One animal had a decreased number of erythrocytes (2,836-2,916 cells per cubic millimetre of blood) and also decreased haemoglobin, haematocrit and iron levels 4.3 μmol/l; [42]. The number of platelets and the differential were all within normal range [42]. Blood chemistry revealed that only two investigated individuals showed increased liver enzymes alkaline phosphatase (ALP) from 424 to 471 U/L and glutamic pyruvic transaminase (GPT) from 109 to 143 U/L compared to reference values of harbour porpoises in human care and from the wild [42].

### Hormone analysis

A summary of the median hormone levels is presented in Table 2.

**Table 2:** Median and first to third quartile of all hormone levels for free-ranging porpoises, porpoises in rehabilitation and in human care (unit for cortisol: ng/ml; for all other hormones: ng/l)

	**Free-ranging**			**Rehabilitation**			**Human care**		
	**n**	**median**	**1. - 3. quartile**	**n**	**median**	**1. - 3. quartile**	**n**	**median**	**1. - 3. quartile**
adrenaline	15	189	126.5-280.5	15	100	82.15-142	11	86.2	58.85-103.24
noradrenaline	15	3068	1970.5-4372	15	1158	993-3040	11	859.25	581.5-1837.5
dopamine	15	294	180.5-448.5	15	61	38.1-465	11	180	150.5-262.7
metanephrine	9	486.6	439.3-589	14	193	109-285.5	2	90	82-98
normetanephrine	9	938.4	676-1107	14	499,5	482.5-611	2	370.5	370.5-422
ACTH	14	283.1	119.8-449.8	9	29,2	20.4-38.4	9	40.4	39.5-58.3
cortisol	14	173.7	120.9-231.4	17	40	27.6-59.9	15	24.3	21.25-31.5

n = number of samples.

#### Adrenaline

**Figure 1:**
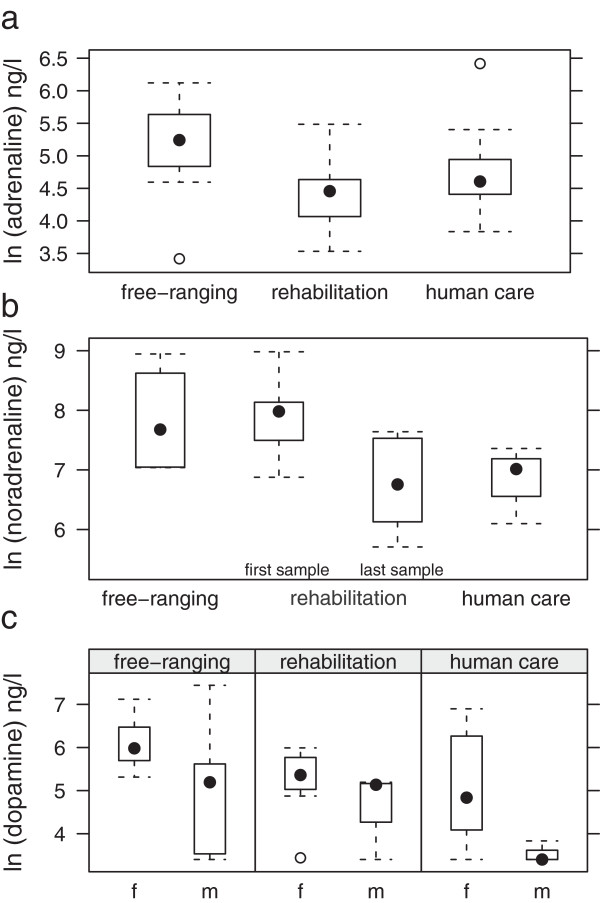
Median levels (ng/l) measured in free-ranging, rehabilitated and permanently kept porpoises (a: adrenalin, b: noradrenaline first and last measurement of rehabilitated animals, c: dopamine for male and female animals).

Median adrenaline levels were significantly higher in free-ranging porpoises than in porpoises in permanent human care (linear mixed model, p = 0.02). Porpoises in rehabilitation showed non-significant, intermediate adrenaline levels (p > 0.1) in comparison to free-ranging animals and animals in human care (Figure 1a). Porpoises in human care (9 animals, 11 samples) showed adrenaline concentrations between 34.2 and 241 ng/l with a median of 86.2 ng/l. Porpoises in rehabilitation (8 animals, 15 samples) ranged between 46 and 612 ng/l. The median of all values was 100 ng/l. In free-ranging porpoises (7 animals, 15 samples) concentrations ranged between 30.5 and 455 ng/l with a median of 189 ng/l.

#### Noradrenaline

Median levels of noradrenaline were significantly higher in free-ranging porpoises (p = 0.0013) and animals in rehabilitation (p = 0.04) when compared to animals in permanent human care using a global comparison with linear mixed models. Porpoises in human care (9 animals, 11 samples) showed noradrenaline concentrations between 301 and 2080 ng/l, the median was 859.3 ng/l.

Noradrenaline values of porpoises in rehabilitation (8 animals, 15 samples) were between 207 and 7,672 ng/l. The median of all values was 1,158 ng/l. The medians of the first and last measurements were 2,591 and 1,112.5 ng/l, respectively. In free-ranging porpoises (7 animals, 15 samples) noradrenaline concentrations of 969–7,954 ng/l were measured, the median was 3,068 ng/l. When splitting the data of the rehabilitated porpoises in two groups by separating the first measurement after arrival in the facility from the last one obtained before release, the first measurement clearly is close to the range of the one found in free-ranging porpoises, while the last one is significantly lower (p = 0.0112) than average measurements from free-ranging individuals (Figure 1b).

#### Dopamine

Dopamine levels did not differ between groups, but females from all groups showed significantly higher values (p = 0.0331) than males (Figure 1c). Porpoises in human care (9 animals, 11 samples) showed dopamine concentrations between 31.2 and 432.8 ng/l, the median was 180 ng/l.

Porpoises in rehabilitation (8 animals, 15 samples) showed values between <30 and 990 ng/l. In 5 of 24 investigated samples dopamine concentration was below the detection limit of 30 ng/l. Dopamine decreased with the length of time animals spent in rehabilitation. In free-ranging porpoises (7 animals, 15 samples) dopamine concentrations of <30–1,708 ng/l were measured, the median was 294 ng/l.

#### Metanephrine

**Figure 2:**
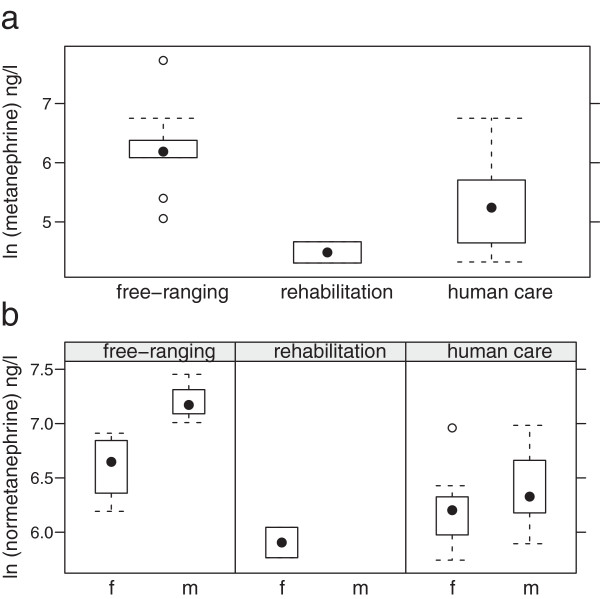
Median levels (ng/l) measured in free-ranging, rehabilitated and permanently kept porpoises (a: metanephrine, b: normetanephrine for male and female animals).

Metanephrine values were significantly higher in free-ranging porpoises compared to animals in human care (p = 0.0176) according to global comparison with linear mixed models (Figure 2a). Values of porpoises in rehabilitation ranged between the two groups. Only samples from two porpoises in human care were analysed, the median was 90 ng/l.

Metanephrine values of porpoises in rehabilitation (5 animals, 14 samples) ranged between 75.4 and 855 ng/l, the median was 193 ng/l. In free-ranging animals (4 animals, 9 samples) metanephrine concentrations between 157 and 2,272 ng/l were measured, the median was 486.6 ng/l.

#### Normetanephrine

Normetanephrine values were significantly higher (p = 0.0124) in free-ranging porpoises than in those in human care (Figure 2b). Values of porpoises in rehabilitation ranged between the two groups. Normetanephrine levels were significantly higher in male porpoises (p = 0.038) throughout all groups.

Two samples from porpoises in human care were analysed, their median was 370.5 ng/l. Normetanephrine values of porpoises in rehabilitation (5 animals, 14 samples) were between 128 and 1,079 ng/l with a median of 499.5 ng/l. In free-ranging animals (4 animals, 9 samples) normetanephrine concentrations between 489 and 1,726 ng/l were found, the median was 938.4 ng/l.

#### ACTH

**Figure 3:**
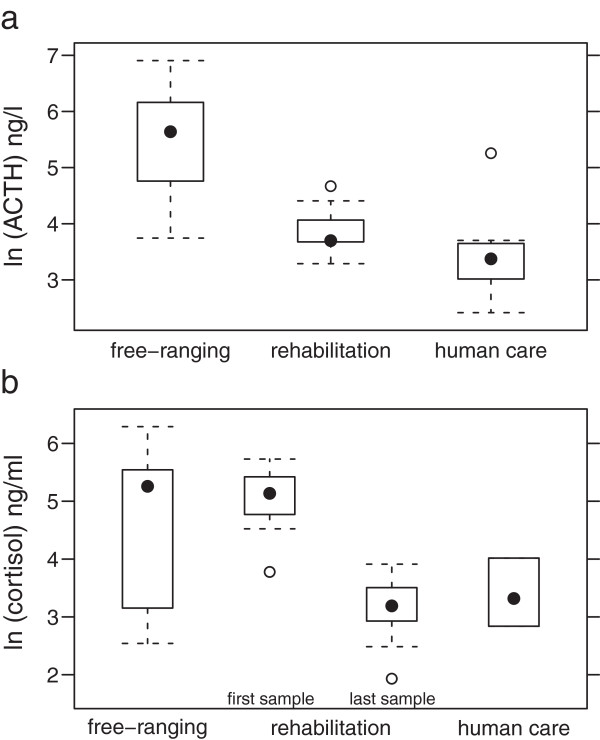
Median levels measured in free-ranging, rehabilitated and permanently kept porpoises (a: ACTH (ng/l), b: cortisol (ng/ml) first and last measurement of rehabilitated animals).

ACTH measurements in porpoises in rehabilitation and human care were significantly lower than those found in free-ranging individuals (p = 0.001; Figure 3a). Porpoises in human care (9 animals, 9 samples) showed concentrations between 26.8 and 82.1 ng/l, the median was 40.4 ng/l. ACTH values of porpoises in rehabilitation (5 animals, 9 samples) were between 11.2 and 191.8 ng/l, the median was 29.2 ng/l. In free-ranging porpoises (7 animals, 14 samples) ACTH concentrations between 42.3- and 998.5 ng/l were measured, the median was 283.1 ng/l.

#### Cortisol

Cortisol levels in porpoises in rehabilitation and human care were significantly lower than in free-ranging individuals (p > 0.001; Figure 3b). In porpoises in human care (9 animals, 15 samples) cortisol concentrations ranged between 6.9 and 54.9 ng/ml, the median was 24.3 ng/ml. Cortisol values of porpoises in rehabilitation (7 animals, 17 samples) ranged between 12.7 and 539 ng/ml, the median was 40 ng/ml. Free-ranging porpoises (7 animals, 14 samples) showed cortisol concentrations between 43.7 and 307.8 ng/ml, the median was 173.7 ng/ml.

When separating the group of rehabilitated porpoises and comparing the first measurement after arrival in the facility with the last one obtained before release, marginally significant differences (p = 0.052) appear between the first value close to the range of the free-ranging animals and the last value similar to the range found in animals in human care (Figure 3b).

### *RT-PCR ana*lyses

A summary of the median mRNA expression levels is presented in Table 3.

**Table 3:** Median and first to third quartile of all mRNA expression levels for free-ranging porpoises, porpoises in rehabilitation and in human care (normalised number of copies)

	**Free-ranging**			**Rehabilitation**			**Human care**		
	**n**	**median**	**1. - 3. quartile**	**n**	**median**	**1. - 3. quartile**	**n**	**median**	**1. - 3. quartile**
HSP70	14	0.60	0.19–1.05	33	0.29	0.19–0.54	19	0.66	0.37–0.95
HP	15	4.66	3.9–7.76	33	4.28	3.4–6.39	19	5.37	3.9–7.2
TNFα	15	0.17	0.14–0.47	32	0.396	0.19–0.83	18	0.42	0.27–0.53
CRP	13	0.005	0.001–0.013	26	0.026	0.005–0.049	19	0.01	0.003–0.011
IL-10	15	0.011	0.01–0.022	26	0.0095	0.005–0.012	13	0.006	0.003–0.009

n = number of samples.

### Acute-phase proteins

#### HSP70

**Figure 4:**
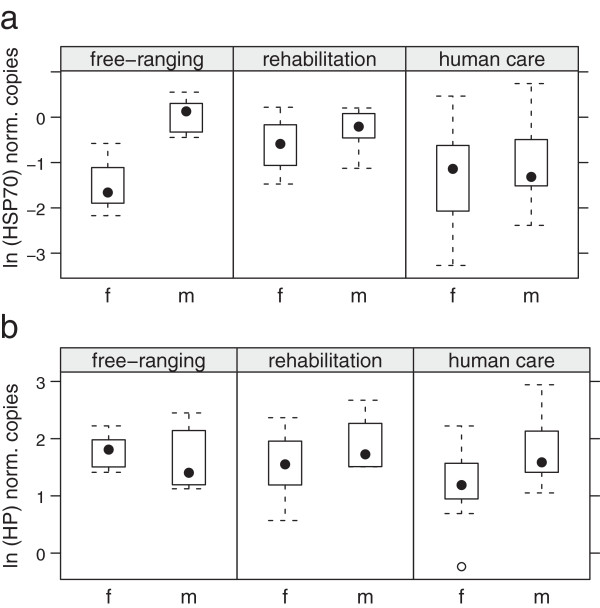
Normalised number of copies measured in male and female free-ranging, rehabilitated and permanently kept porpoises of a): HSP70 mRNA transcripts and b): Hp mRNA transcripts.

mRNA expression levels of HSP70 were significantly higher in male porpoises throughout the groups (p = 0.014; Figure 4a), and porpoises in rehabilitation showed lower values than animals in human care. The first sample taken during the rehabilitation process showed significantly lower ranges than those observed in all other groups (p < 0.05), while the last sample showed values similar to those observed in animals in human care and in the wild.

The median number of HSP70 mRNA transcripts was highest in porpoises in human care (0.655 norm. copies), followed by free-ranging animals (0.601 norm. copies). The lowest number of HSP70 transcripts was found in animals in rehabilitation (0.285 norm. copies).

#### HP

HP expression levels were significantly higher in male porpoises in all groups (p = 0.008; Figure 4b). The median number of HP mRNA transcripts was highest in porpoises in human care (5.37 norm. copies), followed by free-ranging animals (4.66 norm. copies). The lowest number of HP mRNA transcripts was found in porpoises in rehabilitation (4.28 norm. copies).

#### CRP

Porpoises in rehabilitation showed highest levels of CRP expression (p = 0.006), but no differences were found between porpoises in human care and free-ranging porpoises or between porpoises in human care and in rehabilitation (p > 0.2).

### Cytokines

#### Interleukin 10

**Figure 5:**
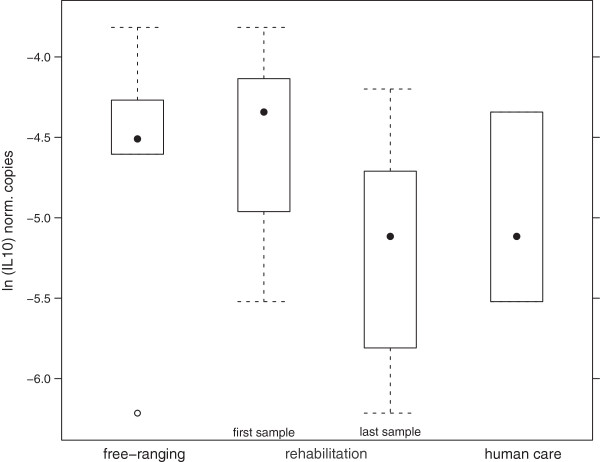
Normalised number of copies of IL10 mRNA transcripts measured in free-ranging, rehabilitated and permanently kept porpoises (first and last measurement of rehabilitated animals).

IL-10 mRNA expression levels were significantly higher in free-ranging porpoises than in animals in human care (p = 0.0196; Figure 5). The median number of IL-10 mRNA transcripts was lowest in porpoises in human care (0.006 norm. copies), followed by individuals in rehabilitation (0.0095 norm. copies). The highest number of IL-10 mRNA transcripts was found in free-ranging animals (0.011 norm. copies).

When separating the individuals from rehabilitation, both first values measured in rehabilitation (median: 0.011 norm. copies) and the values of free-ranging animals were significantly different from individuals in human care (p < 0.05), while the last sample showed values (median: 0.006 norm. copies) similar to those measured in individuals in human care.

#### TNFα

TNFα showed no significant differences between groups (p = 0.14) and sexes (p > 0.4). However, TNFα was correlated with CRP (rho = 0.81, p < 0.001).

#### Correlations between hormones and molecular biomarkers

Correlations were found in stress hormone and mRNA expression levels: IL-10 (rho = 0.44, p = 0.0509) and haptoglobin (rho = 0.59, p = 0.0039) showed significant correlations with normetanephrine. Haptoglobin correlated with metanephrine (rho = 0.64, p = 0.001). There was a positive correlation between IL-10 and cortisol (rho = 0.46, p = 0.004).

## Discussion

Information about hormone status in harbour porpoises is still scarce compared to other marine mammals [11]. Comprehensive studies regarding levels of a broad spectrum of hormones in harbour porpoises have started recently and first results were published in a pilot study [9]. Most of the research performed has focused on thyroid hormones and cortisol [12,16,42]. Although cortisol is one of the most important hormones in stress reaction, its use as a single biomarker of stress is problematic because it does not permit a full assessment of stress situations [22,43]. The present study provides insights in changes in hormone levels as well as in expression levels of immunorelevant molecular biomarkers of harbour porpoises in different stress situations. Capture-induced stress and animal health may be reflected by different markers and may not be related. But the complex mechanisms of the immune and stress response may cause parameters to interact and influence each other, like IL-10 and cortisol in this study.

### Catecholamines

Only little is known about the function of catecholamines as neurotransmitters and hormones in marine mammals. They are associated with an acute fight-or-flight response and their secretion increases rapidly in response to a stressor [11,19]. Median levels of the catecholamines adrenaline, noradrenaline and dopamine estimated in this study were higher in free-ranging harbour porpoises compared to porpoises in human care. Those differences were significant for adrenaline and noradrenaline, most likely caused by the stressful situation for the free-ranging porpoises during capture and handling [9]. The lower median levels in harbour porpoises in human care and at the end of rehabilitation might be explained by an adaptation of those animals to the repeated procedure of sampling. A comparison of catecholamine levels of free-ranging porpoises measured in this study with levels measured by [9] resulted in values three times higher for adrenaline and dopamine and even eight times higher for noradrenaline in the present study. Captive belugas (*Delphinapterus leucas*) exposed to playbacks of high amplitude noise from oil drilling rigs showed little or no discernible effect on adrenaline and noradrenaline concentrations [44], whereas a significant increase in adrenaline, noradrenaline and dopamine concentrations in captive belugas exposed to sound was observed by other researchers [10]. Although no firm conclusions can be drawn due to limited sample size, the findings correspond with those of other authors [10,11] and indicate that catecholamines can be used as markers of stress in harbour porpoises.

### Cortisol and adrenocorticotropic hormone

Glucocorticoids such as cortisol have many physiological functions in metabolism and the nervous and cardiovascular systems. They have an immunosuppressive and anti-inflammatory effect. The release of ACTH is mediated by corticotrophin-releasing hormone (CRH). Under stress, ACTH is released in a pulsatile fashion [45] and is responsible for the stimulation of cortisol secretion in the adrenal cortex. The median levels of ACTH and cortisol for free-ranging porpoises estimated in this study were significantly higher than those for porpoises in rehabilitation and in human care. This was observed earlier in a study in which free-ranging porpoises showed ACTH concentrations five times higher than individuals in human care [9]. Animals in human care taken out of the water for blood sampling were shown to have higher levels of cortisol than animals presenting their fluke for a voluntary blood sample [16]. The cortisol levels of free-ranging porpoises in the present study were similar to those measured in porpoises during tagging [12]. Nevertheless, it has to be taken into account that the range of measured cortisol levels was highly variable in all investigated groups, indicating an individual response to the same treatments. The fact that the cortisol levels estimated at the beginning of rehabilitation were close to the range of the free-ranging animals and the levels at the end of rehabilitation were similar to the range in animals in human care indicates a habituation of the animals to the handling procedure. Interestingly, the cortisol levels in the present study were close to the levels measured in blood samples voluntarily given by porpoises in human care [16].

### Metanephrine and normetanephrine

These parameters, as methylation products of adrenaline and noradrenaline, were used as more stable equivalents, but a tendency or change in values between the groups or a correlation between metanephrine and adrenaline as well as between normetanephrine and noradrenaline values did not appear, probably because sample sizes for estimating metanephrine and normetanephrine were much smaller than for adrenaline and noradrenaline. Multiple sampling in one porpoise in rehabilitation produced correlating adrenaline/metanephrine and noradrenline/nor-metanephrine gradients. This may be due to the longer half-life of catechalomines in marine mammal metabolism [10,11]. In free-ranging animals multiple samples were taken within several hours when degradation products had not yet been produced, while samples from porpoises in rehabilitation were taken over a period of several days. The correlations between metanephrine and normetanephrine and acute-phase protein biomarkers (HP, HSP70) indicate that these markers are expressed in stress responses.

### APP

#### HSP70

The synthesis of HSP70 can be induced by heat, lack of glucose, hyperosmolarity, hypoxia or ischemia [46]. As cell mediator HSP70 transfers unspecific stress information to the innate and adaptive immune system [47]. Upregulation of HSP70 constrains secretion of TNFα [48]. Expression levels of HSP70 in this study were highest in porpoises in human care, followed by free-ranging animals. The lowest number of HSP70 transcripts was found in porpoises in rehabilitation, whereas TNFα and CRP were highest in rehabilitation. Values of first samples taken from animals in rehabilitation were significantly lower than in porpoises from the other groups (p < 0.05). Female porpoises showed significantly lower HSP70 expression levels than males. It has been reported that male rats show higher expressions of heat-shock protein genes (e.g. HSP70) suggesting that cellular stress is elevated in males [49]. Other studies on mammals have shown that HSP70 expression is induced by exercise - possibly correlated to the findings in this study where diseased animals in rehabilitation showed lowest levels - and that this effect is attenuated by female hormones [50]. Sex-related differences in APP expression levels show the biological differences between sexes and indicate the wide physiological impact of hormones on the metabolism.

#### HP

Highest ranges were measured in porpoises in human care, probably induced by elevated TNFα levels [51].

An increase in HP concentrations can be caused by inflammation and infectious diseases and is associated with physical, psychological or environmental stress. HP values varied strongly in stable Steller Sea lion (*Eumetopias jubatus*) and harbour seal (*Phoca vitulina*) populations in the region of Alaska when compared to other populations undergoing a strong population decrease over several years [14]. High HP expression was described for harbour porpoises correlating with increased leukocytes [33]. However, the same was not observed in the free-ranging porpoises of the present study.

Female porpoises showed significantly lower HP expression levels than males. This was also observed for HSP70 and could reflect hormone attenuated or sex-related differences in APP expression.

#### CRP

The correlation found between CRP and TNFα probably shows that both markers interact physiologically and that animals in rehabilitation, with highest values, were diseased.

### Cytokines

#### IL-10

Significantly higher IL-10 values (p = 0.0196) were found in free-ranging porpoises than in those in human care. Elevated IL-10 levels may indicate a continuous stimulation of the immune system due to infectious agents [52] in porpoises from the wild. IL-10 is an anti-inflammatory cytokine and inhibits several immune responses, e.g. the production of pro-inflammatory cytokines such as TNFα [53,54]. The correlation between IL-10 and cortisol observed in this study may indicate higher stress levels and more exposure to infectious disease in free-ranging porpoises.

#### TNFα

TNFα belongs to the early immune response of pro-inflammatory cytokines, and induces APP. The correlation found between TNFα and CRP in this study probably reflects this mechanism and also shows that both markers may be expressed as a sign of disease, as porpoises in rehabilitation showed highest values.

Indication for induction of APPs by TNFα was also found in both HP and HSP70 that showed high values in porpoises in human care. Although no significant differences were found between groups, a clear trend for lowest numbers of TNFα transcripts in free-ranging porpoises and for highest numbers in porpoises in human care was apparent. Glucocorticoids and catecholamines are supposed to constrain secretion of TNFα [55]. Low levels of TNFα in free-ranging porpoises may be caused by constraint due to stress hormones, but as the endocrine and immune systems are complex systems this is difficult to determine.

## Conclusions

In conclusion, profiles of stress hormones can be a useful tool for the determination of stress in harbour porpoises. Stress hormone levels are significantly higher in free-ranging porpoises compared to animals in human care as well as compared to animals in rehabilitation when splitting those measured values in first and last sample during the rehabilitation period. This decrease of hormone levels due to habituation to human handling demonstrates the importance of investigations on animals during the rehabilitation process.

Hormone levels of free-ranging harbour porpoises and animals at the beginning of rehabilitation can be seen as base values for stressed porpoises, levels of porpoises in human care and at the end of rehabilitation as base values for less stressed, habituated porpoises, possibly equalling the baseline values of wild healthy porpoises. Nevertheless, the variation in measured values, especially in free-ranging porpoises, has to be taken into account. Diverse factors might explain this variation, e.g. the natural variation among the wild species, different age categories or unknown variation in life history and environmental conditions. The investigation of the mRNA expression of the immunorelevant biomarkers reflects the development of the immune status over time and therefore serves as a sensible indicator for the effect of stress on the early immune response. The positive correlations observed in this study (e.g. IL-10 and cortisol) demonstrate the dynamic interaction between immune response and stress response. Due to the complexity of the immune and endocrine systems, further investigations on harbour porpoises in permanent or temporary human care under controlled and repeated conditions are needed to unravel the remaining uncertainties.

## Abbreviations

CRH: Corticotropin-releasing hormone; ACTH: Adrenocorticotropic hormone; mRNA: Messenger-Ribonucleic acid; HSP70: Heat shock protein 70; IL-10: Interleukin10; TNFα: Tumor necrosis factor a; n: number; EDTA: Ethylenediaminetetraacetic acid; C: Celsius; EGTA: Ethyleneglycoltetraacetic acid; HPLC: High-performance liquid chromatography; ECLIA: Electrochemiluminescent immunoassay; UK: United Kingdom; GAPDH: Glyceraldehyde 3-phosphate dehydrogenase; APP: Acute-phase proteins; CRP: C-reactive protein; YWHAZ: 14-3-3 proteinzeta/delta; HP: Haptoglobin; RT-PCR: Real-time polymerase chain reaction; PCR: Polymerase chain reaction; LMM: Linear mixed models; Porpoise ID: Identification number of individual porpoises; ID: Identification; AIC: Akaikes Information Criterion; ALP: Alkaline phosphatase; GPT: Glutamic pyruvic transaminase; Fig: Figure; Tab: Table; DK: Denmark; NL: The Netherlands.

## Competing interests

The authors declare that they have no competing interests.

## Authors’ contributions

JD, EE, JK, CvN, SM and JT conducted sampling and preparation of samples. SM, HS, JD, US and KL conducted analyses. KR conducted the statistical analyses. SM, KL, KR, JD and US drafted the manuscript and the other authors contributed to the manuscript. All authors read and approved the final manuscript.
